# The Role of Water in Activation Mechanism of Human N-Formyl Peptide Receptor 1 (FPR1) Based on Molecular Dynamics Simulations

**DOI:** 10.1371/journal.pone.0047114

**Published:** 2012-11-26

**Authors:** Shuguang Yuan, Umesh Ghoshdastider, Bartosz Trzaskowski, Dorota Latek, Aleksander Debinski, Wojciech Pulawski, Rongliang Wu, Volker Gerke, Slawomir Filipek

**Affiliations:** 1 International Institute of Molecular and Cell Biology, Warsaw, Poland; 2 Nencki Institute of Experimental Biology, Polish Academy of Sciences, Warsaw, Poland; 3 Faculty of Chemistry, University of Warsaw, Warsaw, Poland; 4 Institute of Medical Biochemistry, Centre for Molecular Biology of Inflammation, University of Münster, Münster, Germany; German Institute of Human Nutrition Potsdam-Rehbruecke, Germany

## Abstract

The Formyl Peptide Receptor 1 (FPR1) is an important chemotaxis receptor involved in various aspects of host defense and inflammatory processes. We constructed a model of FPR1 using as a novel template the chemokine receptor CXCR4 from the same branch of the phylogenetic tree of G-protein-coupled receptors. The previously employed template of rhodopsin contained a bulge at the extracellular part of TM2 which directly influenced binding of ligands. We also conducted molecular dynamics (MD) simulations of FPR1 in the apo form as well as in a form complexed with the agonist fMLF and the antagonist tBocMLF in the model membrane. During all MD simulation of the fMLF-FPR1 complex a water molecule transiently bridged the hydrogen bond between W254^6.48^ and N108^3.35^ in the middle of the receptor. We also observed a change in the cytoplasmic part of FPR1 of a rotamer of the Y301^7.53^ residue (tyrosine rotamer switch). This effect facilitated movement of more water molecules toward the receptor center. Such rotamer of Y301^7.53^ was not observed in any crystal structures of GPCRs which can suggest that this state is temporarily formed to pass the water molecules during the activation process. The presence of a distance between agonist and residues R201^5.38^ and R205^5.42^ on helix TM5 may suggest that the activation of FPR1 is similar to the activation of β-adrenergic receptors since their agonists are separated from serine residues on helix TM5. The removal of water molecules bridging these interactions in FPR1 can result in shrinking of the binding site during activation similarly to the shrinking observed in β-ARs. The number of GPCR crystal structures with agonists is still scarce so the designing of new ligands with agonistic properties is hampered, therefore homology modeling and docking can provide suitable models. Additionally, the MD simulations can be beneficial to outline the mechanisms of receptor activation and the agonist/antagonist sensing.

## Introduction

Human N-formyl peptide receptors (FPRs) are G protein-coupled receptors (GPCRs) involved in many physiological processes, including host defense against bacterial infection and resolving inflammation [1]–[8]. The three human FPRs (FPR1, FPR2 and FPR3) share significant sequence homology and perform their action via coupling to G_i_ protein. Activation of FPRs induces a variety of responses, which are dependent on the agonist, cell type, receptor subtype, and also species involved. FPRs are expressed mainly by phagocytic leukocytes. Together, these receptors bind a large number of structurally diverse groups of agonistic ligands, including *N*-formyl and nonformyl peptides of different composition, that chemoattract and activate phagocytes. For example, N-formyl-Met-Leu-Phe (fMLF), an FPR1 agonist, activates human phagocyte inflammatory responses, such as intracellular calcium mobilization, production of cytokines, generation of reactive oxygen species, and chemotaxis [9]. This ligand can efficiently activate the major bactericidal neutrophil functions and it was one of the first characterized bacterial chemotactic peptides [10]. Whereas fMLF is by far the most frequently used chemotactic peptide in studies of neutrophil functions, atomistic descriptions for fMLF-FPR1 binding mode are still scarce mainly because of the absence of a crystal structure of this receptor. Elucidating the binding modes may contribute to designing novel and more efficient non-peptide FPR1 drug candidates. Molecular modeling of FPR1, on the other hand, can provide an efficient way to reveal details of ligand binding and activation of the receptor. However, recent modeling studies of FPRs were confined only to bovine rhodopsin [11], [12] as a template.

Recently, Fujita *et al.* [13] investigated binding of calpain inhibitors as well as short peptides including fMLF to FPR1 and FPR2 receptors. Their findings suggest that potent calpain inhibitors could stimulate phagocyte functions via activation of FPR1, FPR2 and/or other G-protein coupled receptors depending on the inhibitors used. Using molecular docking they obtained different binding modes of fMLF in the above receptors and compared qualitatively the estimated energies of ligand binding to the experimental data. They also provided a list of residues in vicinity of the ligand but they did not show ligand-receptor interactions in the binding site. In another paper, Khlebnikov *et al.* [14] investigated binding of a set of benzimidazole derivatives as well as other agonists of FPR1 including fMLF. After the docking the 2 ns molecular dynamics (MD) simulations confined to the binding site were conducted. The rest of the FPR1 structure was kept rigid. In the best scored pose of fMLF-FPR1 the C-terminus of the ligand interacted with R205^5.42^ while the formylated N-terminus interacted with the main chains of residues L198^5.35^-V200^5.37^ which could suggest that this part of the helix was unfolded. In another report Movitz *et al.* [15] identified the shortest sequence of the FPR1 ligand annexin A1 [16] which was still able to activate FPR1 and they also investigated the binding modes of this tetrapeptide. The Gln^9^-Phe^12^ (Ac-QAWF) peptide was the shortest peptide of annexin A1 possessing the capacity both to trigger a neutrophil NADPH oxidase response and to inhibit the activity induced by other FPR agonists. Two alternative binding modes of Ac-QAWF were found having the same position of the N-terminus close to residues D106^3.33^, R201^5.38^ and R205^5.42^. However, in neither configuration there was interaction with R86^2.65^ which was predicted to be a part of the binding site for fMLF based on mutagenesis experiments [17]. In all the above studies the rhodopsin structure was taken as a template and no molecular dynamics simulations of the receptor in the membrane were performed to investigate an influence of the ligand on the receptor structure.

To locate specific ligand-receptor interactions based on a more appropriate template than rhodopsin we generated the homology models of FPR1 using the crystal structure of the chemokine receptor CXCR4 [18], which shares over 30% sequence identity with FPR1 and is located in the same γ branch of the phylogenetic tree of GPCRs (gpcr.scripps.edu). Docking and model refinement procedures were pursued afterward. Nine 100 ns full-atom MD simulations in three repeats were conducted for the Apo form as well as for complexes of fMLF (agonist) and tBocMLF (antagonist) with FPR1 in the membrane. Based on locations of the N- and C-termini of the ligand the FPR1 extracellular pocket can be divided into two zones, namely, the anchor and activation regions. The formylated M1 residue of fMLF bound to the activation region led to a series of conformational changes of conserved residues. Internal water molecules participating in extended hydrogen bond networks were found to play a crucial role in transmitting the agonist-receptor interactions. A mechanism is proposed concerning the initial steps of receptor activation concurrent with ligand binding.

## Results

### FPR1 structure and the binding pocket

**Figure 1:**
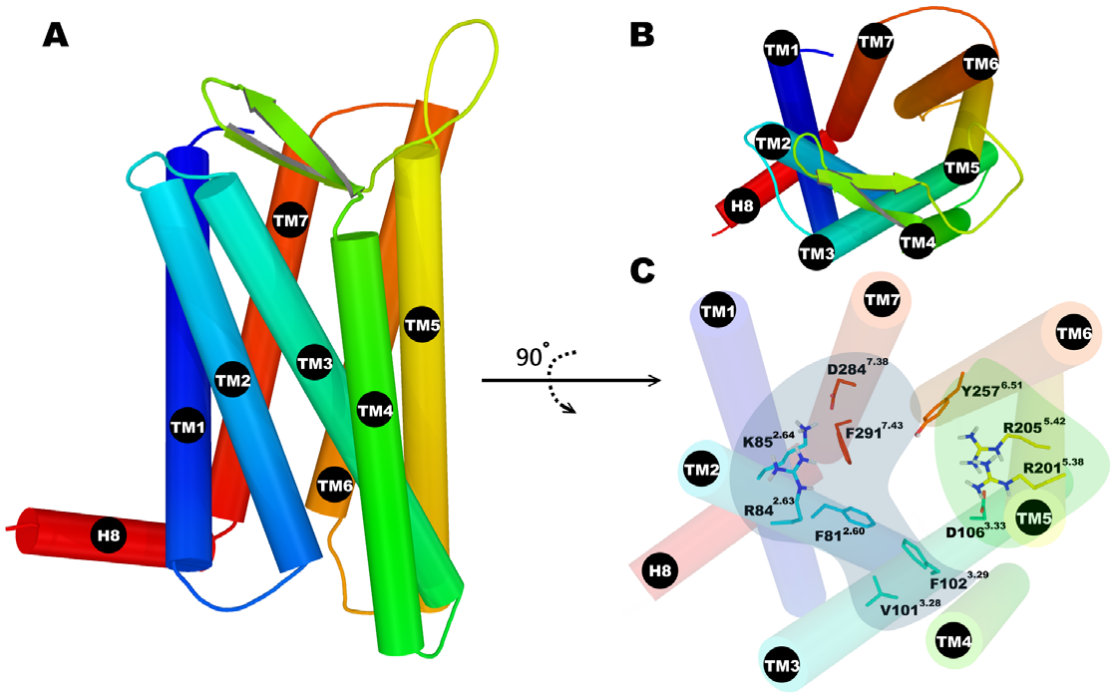
The structure of homology model of FPR1 and its binding pocket. (**A**) overall view of FPR1 model; (**B**) alternative view of FPR1 model from extracellular side; (**C**) important residues in binding site of FPR1. The whole pocket was visually divided into two zones: the anchor region (on the left - in blue) and the activation region (on the right – in green).

**Figure 2:**
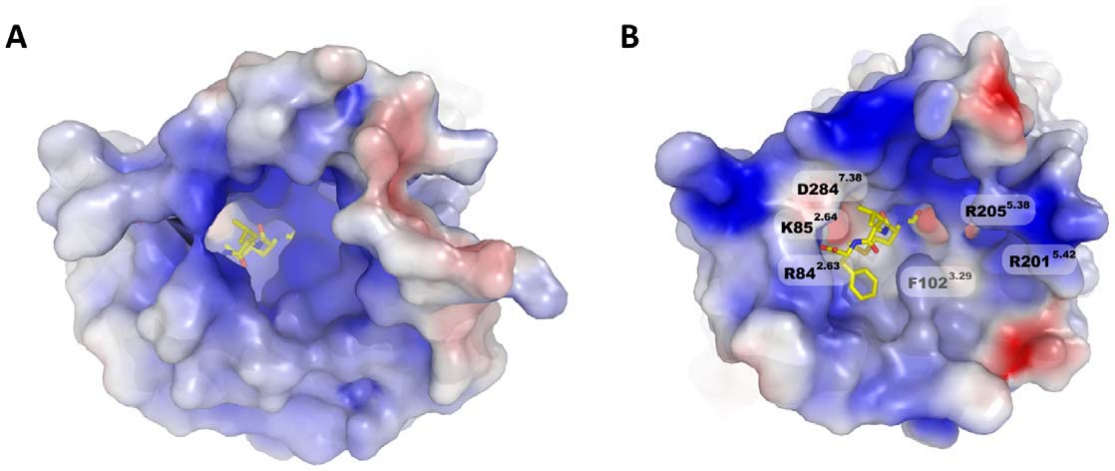
Extracellular surface of FPR1 model after equilibration period. The structure is mapped with electrostatic potential (positive in blue, negative in red) and a position of agonist is shown. (**A**) The whole structure of the model. (**B**) The model without all extracellular loops. Selected important and visible on molecular surface residues are labeled.

**Figure 3:**
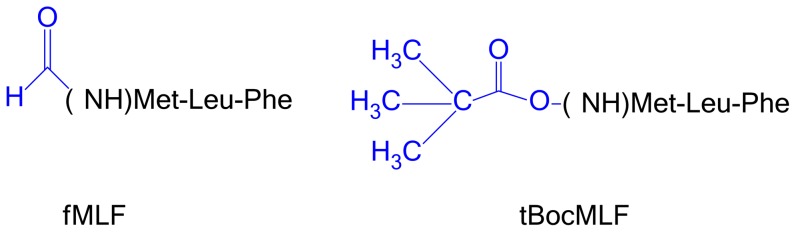
The chemical formulas of fMLF (agonist) and tBocMLF (antagonist). Both ligands share most of the structure so only differences in N-termini are shown in detail and colored in blue.

Currently, in the γ branch of the most populated family A of GPCRs there are five receptors whose structure has been determined, the chemokine receptor CXCR4 [18], the opioid receptors: μOR [19], δOR [20], κOR [21] and the nociceptin FQ receptor [22]. For the homology modeling of FPR1 we used the one most similar in sequence and the closest in the phylogenetic tree, the chemokine receptor. The model obtained for the FPR1 structure consists of a seven transmembrane (TM) helix bundle (TM1 to TM7), a cytosol helix H8 and a β-hairpin loop between TM4 and TM5 (Figure 1A and 1B). Although the structure of CXCR4 does not contain helix H8 it exists in all crystal structures of opioid receptors which suggests that H8 is unfolded in the crystal of CXCR4 because of crystal packing. The model of FPR1 was relaxed in a POPE membrane using detailed relaxation procedure in Desmond program (see Methods section) and subjected to ligand docking. The fMLF binding site of modeled FPR1 is quasi symmetrical (Figure 1C). At both ends of the binding site there are positively charged residues: R84^2.63^ and K85^2.64^ located in TM2 as well as R201^5.38^ and R205^5.42^ on helix TM5. They are complemented by negatively charged residues: D284^7.38^ in TM7 interacting with K85^2.64^ and, at the other end, D106^3.33^ in TM3 interacting with R201^5.38^. However, D106^3.33^ is located much deeper in the receptor structure than D284^7.38^ and is tightly interacting with R201^5.38^. Between both areas there are hydrophobic residues separating these charged areas and also interacting with the ligand. They can also be divided into two zones: residues F81^2.60^, V101^3.28^ and F102^3.29^ on helices TM2 and TM3 are located on one side of the ligand whereas Y257^6.51^ and F291^7.43^ on helices TM6 and TM7 on the other side. All abovementioned residues are located within 4 Å of the ligand. Because of such distribution of residues the entrance to the binding site is nearly uniformly positively charged (Figure 2) so that the negatively charged ligands will be selectively attracted. As for the agonist fMLF, and antagonist, tBocMLF, (Figure 3) they would enter the binding site most preferably with the negatively charged C-terminus. The residue D106^3.33^ is buried under R201^5.42^ and is not visible in Figure 2B. The red spot of negative potential in the center of the receptor (Figure 2B) comes from residue N108^3.35^. To facilitate comparison of our structure to other GPCRs the Ballesteros-Weinstein numbering scheme [23] was used (numbers in superscript) apart from the sequence numbers of FPR1 residues.

### Interactions of ligands with binding site of FPR1

**Figure 4:**
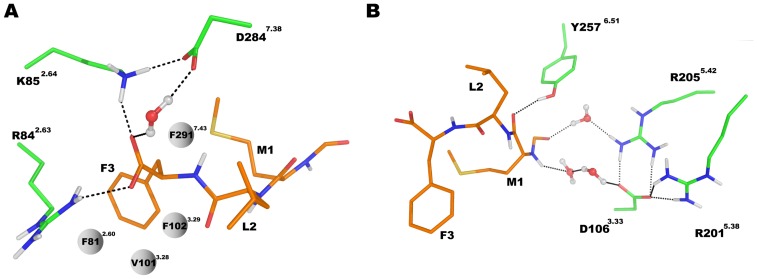
The structure of agonist fMLF interacting with FPR1 after equilibration period. (**A**) Interactions between C-terminus of fMLF with FPR1. The residues R84^2.63^ and K85^2.64^ were found to form direct salt bridges with carboxyl terminus of ligand while D284^7.38^ interacts with the same group of agonist through a water molecule. Hydrophobic side chain of fMLF is surrounded by F81^2.60^, V101^3.28^, F102^3.29^ and F291^7.43^. (**B**) Interactions between N-terminus of fMLF and FPR1. The carbonyl group in peptide bond in residue M1 forms a direct hydrogen bond with Y257^6.51^ while the formyl group can interact with both R205^5.42^ and D106^3.33^ through water molecules.

**Figure 5:**
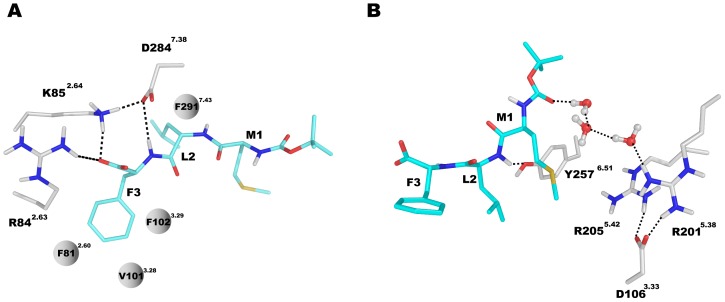
The structure of antagonist tBocMLF interacting with FPR1 after equilibration period. (**A**) Interactions between C-terminus of tBocMLF with FPR1. The residues R84^2.63^, K85^2.64^ and D284^7.38^ form hydrogen bonds with tBocMLF directly. Hydrophobic side chains of antagonist are surrounded by F81^2.60^, V101^3.28^, F102^3.29^ and F291^7.43^. (**B**) Interactions between N-terminus of tBocMLF and FPR1.

The three best scored binding configurations (poses) out of the 2000 conformations for agonist and antagonist, respectively, were characterized by the C-terminus of both ligands bound to the charged area at TM2 (the anchor region) whereas the N-terminus of the ligand was bound to the second charged area at TM5 (the activation region). The same hydrophobic residues of both ligands (Figure 3) can suggest similar preferential binding modes. Next we conducted equilibration calculations of both complexes in a model of POPE membrane. After equilibration the C-terminal residue F3 of the agonist was engaged in a stable hydrogen bond network (Figure 4A) formed by the side chains of R84^2.63^, K85^2.64^ and D284^7.38^ while a water molecule mediated the hydrogen bonds between fMLF carbonyl group and D284^7.38^. The side chain of residue F3 was surrounded by four hydrophobic residues, namely F81^2.60^, V101^3.28^, F102^3.29^ and F291^7.43^. Similarly to the agonist in the C-terminal region, the antagonist tBocMLF also formed hydrogen bonds directly with R84^2.63^ and K85^2.64^ (Figure 5A), while the side chain of F3 was also stabilized by hydrophobic residues F81^2.60^, V101^3.28^, F102^3.29^ and F291^7.43^. Differently from the agonist a hydrogen bond of tBocMLF with D284^7.38^ was not created or even bridged by a water molecule but instead the NH group of the peptide bond in residue F3 formed a hydrogen bond with D284^7.38^ directly.

At the other end of the two ligands the N-terminal formyl group of the agonist (Figure 4B) was involved in a complex water-mediated hydrogen bond network including residues R205^5.42^ and D106^3.33^ while the carbonyl group of the peptide bond in residue M1 formed a hydrogen bond with Y257^6.51^. Similarly to the agonist no direct interactions with charged residues of the receptor were found in the N-terminus of tBocMLF and only a water mediated hydrogen bond network was located between the carbonyl group of tBoc and two arginine residues R201^5.38^ and R205^5.42^ (Figure 5B). Moreover, there was also a direct hydrogen bond between Y257^6.51^ and the main chain of the antagonist.

### MD simulations

To investigate the changes in FPR1 structure that can be induced concurrently with agonist binding we performed 100 ns MD simulations starting from systems equilibrated in a model membrane. The simulations were conducted for FPR1 in its Apo form, as well as for complexes with agonist and antagonist. The root mean squares deviation (RMSD) plots of the protein backbone show small rearrangements (0.7 Å) compared to the starting structures so the investigated structures were stable as early as 5 ns after MD simulation started (Figure S1 in File S1) indicating that the equilibration procedure was sufficient to stabilize the receptor. The binding pocket remained similar to the starting conformations in all conducted simulations suggesting that only local rearrangements took place at least at this time-scale. Each simulation was repeated three times with different seeds and the final structures for a repeated round of each case are similar to each other.

**Figure 6:**
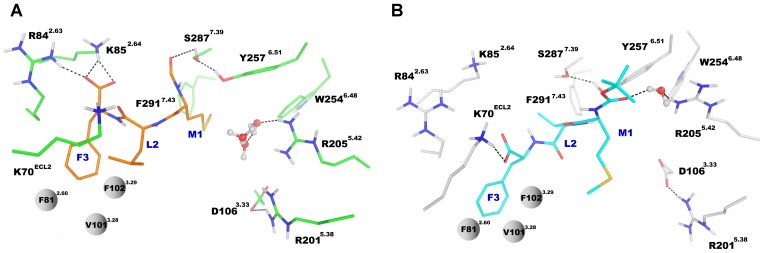
The ligand-receptor interactions after 100 ns MD simulation. View from extracellular side. (**A**) The agonist fMLF (in orange). (**B**) The antagonist tBocMLF (in cyan). The M1 residue of agonist went down toward W254^6.48^ while that of antagonist went up toward EC2 loop.

During the simulations both agonist and antagonist changed their positions, however, the agonist stayed bound to the anchor region for the whole simulation while the antagonist moved and finally its charged C-terminus interacted directly with K170 from a long EC2 loop (between TM4 and TM5). Additionally the benzene ring of F3 of antagonist formed π-π stacking interactions with W91 of the EC1 loop. In the case of agonist the side chain of F3 was stably located between F81^2.60^, W91^EC1^ and F102^3.29^ (Figure 6A). At the N-terminus of the antagonist there was a large movement of residue M1 from an interior position toward EC2 and especially residue F178. The tBoc group did not change much its position but a hydrogen bond to Y257^6.51^ was lost (Figure 6B). In the case of agonist there was also a change of the M1 side chain but here towards the interior of FPR1 close to the position previously occupied by the formyl group of this ligand i.e. close to residues R205^5.42^, Y257^6.51^ and W254^6.48^. M1 also displaced one water molecule and stayed close to F291^7.43^. The formyl group interacted with S287^7.39^ and indirectly with Y257^6.51^ (Figure 6A). The electrostatic interactions between D106^3.33^ and both arginine residues, R201^3.38^ and R205^5.42^, were stable in the Apo form of FPR1 (Figures S2A and S3A in File S1). However, for both the antagonist and agonist the interaction D106^3.33^-R201^3.38^ was broken and restored many times (Figure S2A in File S1). For both ligands the residue R205^5.42^ moved away from D106^3.33^ but in the case of agonist it was separated by only one water molecule (Figure S2B in File S1).

**Figure 7:**
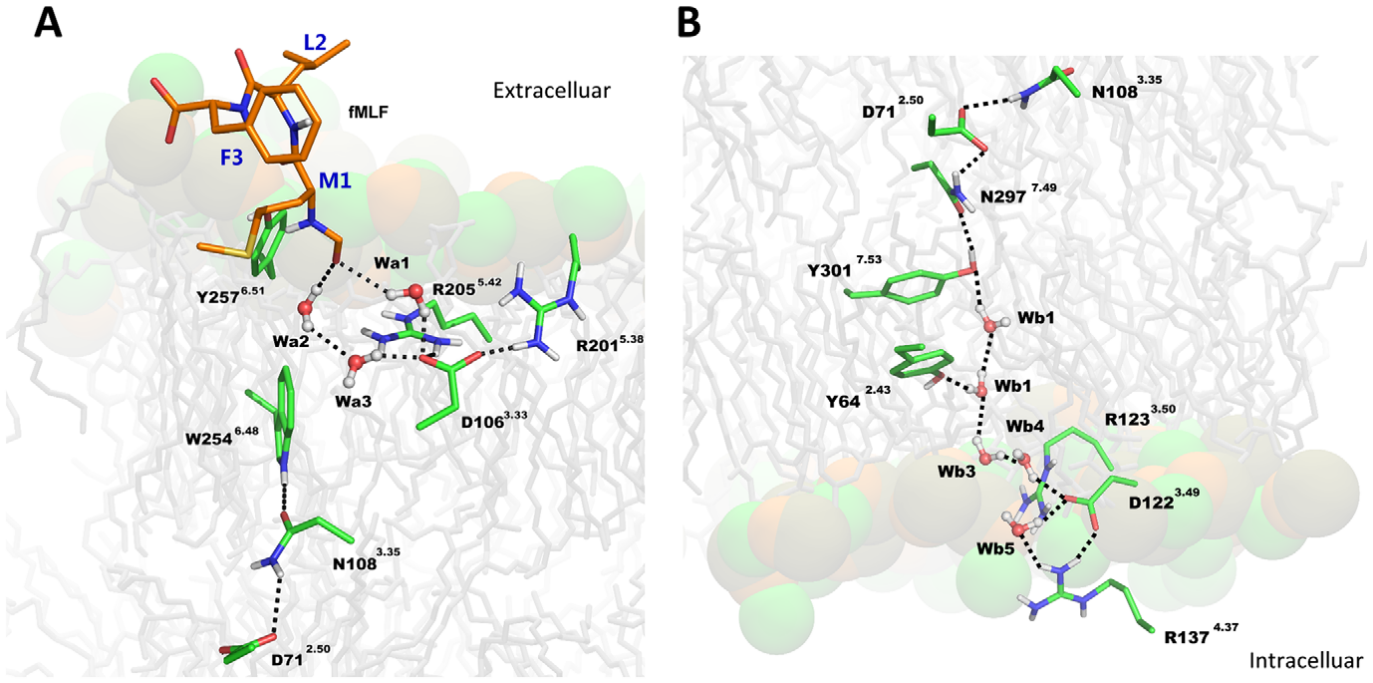
A hydrogen bond network in the structure of the agonist-FPR1 complex. A side view of initial equilibrated structure. (**A**) The binding site showing the hydrogen bond network involving water molecules. (**B**) A continuation of the hydrogen bond network of the same complex at the intracellular side.

**Figure 8:**
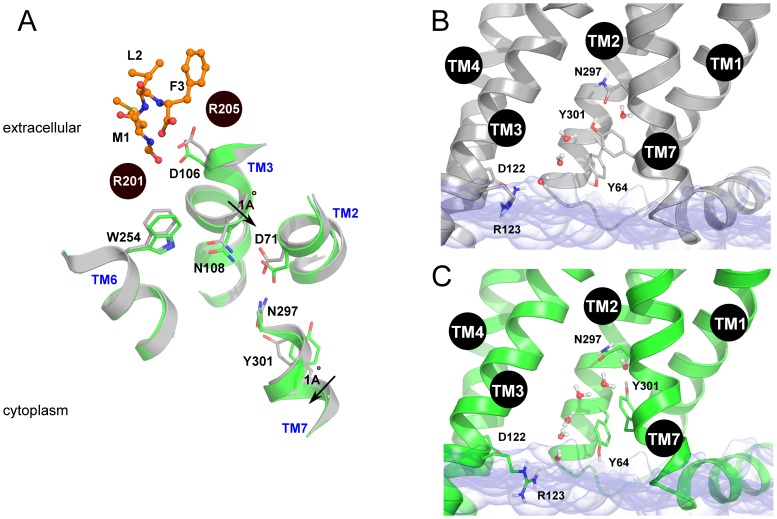
Mechanism of partial activation of FPR1. (A) Movement of helices due to agonist binding. Apo structure in gray and with agonist bound in green; (B) structure of cytoplasmic part of FPR1 in Apo form and with antagonist bound; (C) structure of cytoplasmic part of FPR1 in agonist bound complex. The hydrogen bond between Y301^7.53^ and N298^7.49^ was found bridged by a water molecule and the residue Y301^7.53^ was switched.

**Figure 9:**
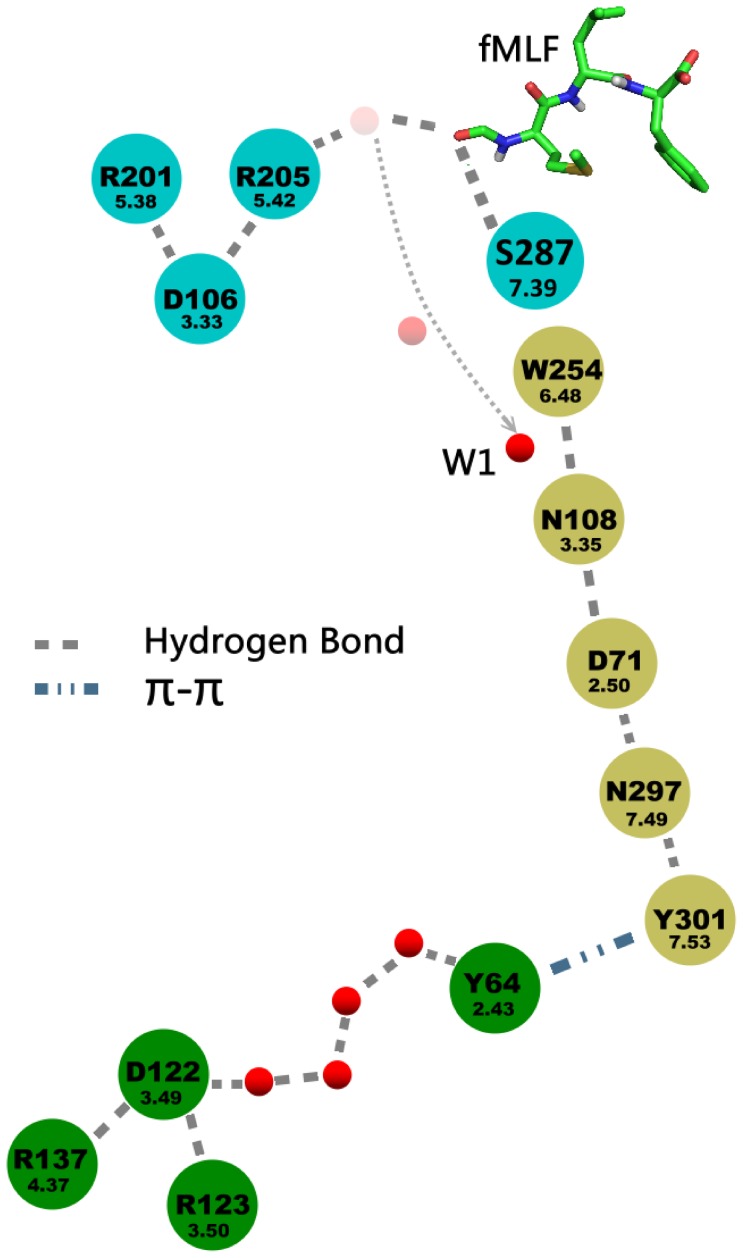
A scheme of interactions in the final structure of the agonist-FPR1 complex after MD simulation. A movement of two water molecules during MD simulation is shown. These molecules can bridge the hydrogen bonds between some residues. A water molecule transiently bridges a hydrogen bond between W254^6.48^ and N108^3.35^.

Similarly to other structures of GPCRs in a partly activated state a hydrogen bond network has been found throughout the whole transmembrane region of FPR1 (Figure 7). This network started from W254^6.48^ and consisted of residues N108^3.35^ (the residue also present in CXCR4 and opioid receptors but not in muscarinic receptors), D71^2.50^ (the most conserved residue in TM2), N297^7.49^ and Y301^7.53^ (of the NPxxY motif). The above residues were connected directly by hydrogen bonds. Y301^7.53^ formed π-π stacking interaction with Y64^2.43^ but also participated in water-mediated hydrogen bond networks involving additionally the residues at the cytoplasmic part of the receptor: Y64^2.43^, D122^3.49^ and R123^3.50^ (from the DRC motif – corresponding to DRY in other GPCRs) as well as R137^4.37^ interacting directly with D122^3.49^ (Figure 7B). During MD simulation of the fMLF-FPR1 complex a water molecule initially located between R205^5.42^ and W254^6.48^ diffused toward the center of FPR1 and transiently bridged the hydrogen bond between W254^6.48^ and N108^3.35^ (Figure S3A in File S1 and Movie S1). At the cytoplasmic end of FPR1 water molecules were present up to the N297^7.49^ residue in all investigated systems (Figure 8B,C). However, in all three simulations of FPR1 with agonist bound we observed a change of a rotamer of the Y301^7.53^ residue (a switch called a tyrosine rotamer toggle switch). It happened at different times (80 ns, 40 ns, and 70 ns) in different simulations with agonist (Figure S3B in File S1). After the rotamer change a water molecule present close to N297^7.49^ was bridging the N297^7.49^-Y301^7.53^ interaction. This bridging was stable till the end of each simulation. The effect of agonist on movement of the surrounding helices is shown in Figure 8A. The network of interactions between residues of the agonist-receptor complex as well as the movement of bridging water molecules is depicted schematically in Figure 9. A hydrogen bond between a formyl group and S287^7.39^ was formed in all MD simulation of FPR1 with agonist (Figure S4 in File S1); this bond can also influence the movement of helices and change of the rotamer switch of Y301^7.53^.

### Models of FPR2 and FPR3 receptors

**Figure 10:**
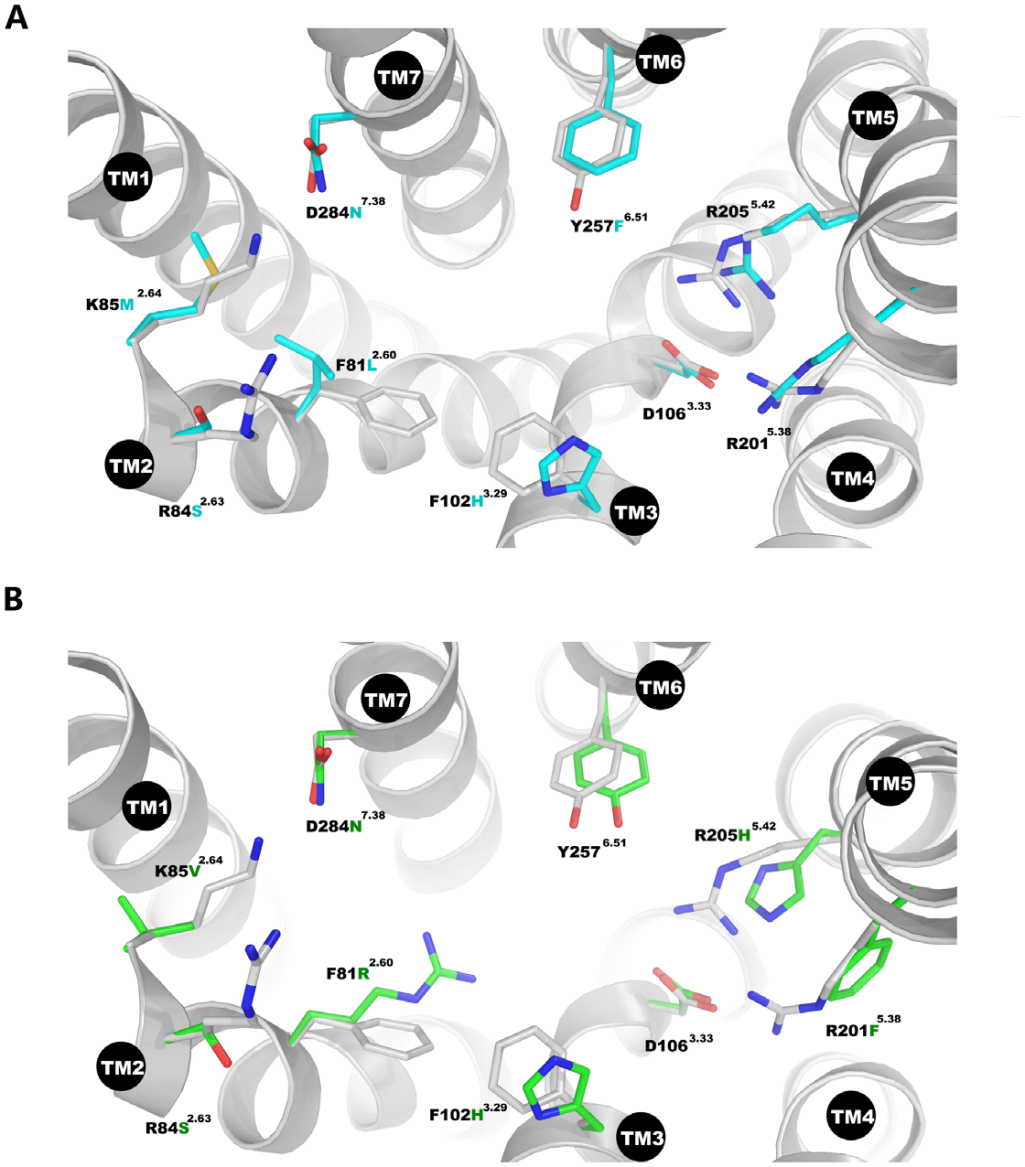
Superimposed models of FPRs constructed using CXCR4 template structure. (**A**) Superimposition of FPR1 (gray) and FPR2 (cyan). (**B**) Superimposition of FPR1 (gray) and FPR3 (green).

To obtain models of the related receptors FPR2 and FPR3 we performed homology modeling based on the equilibrated structure of FPR1. The FPR2, which shares 69% sequence identity with FPR1, is a low affinity receptor for fMLF with a K_d_ of 430 nM [24]–[26]. The obtained model of FPR2 showed many differences compared to FPR1 including residues in the binding site: (FPR1 to FPR2) F81L^2.60^, R84S^2.63^, K85M^2.64^, F102H^3.29^, Y257F^6.51^ and D284N^7.38^ (Figure 10A). Since K85^2.64^ and R84^2.63^ has been experimentally proven to be crucial for fMLF binding [17], the mutations at these two positions in FPR2 may be responsible for the low binding affinity of fMLF. We also performed docking of this agonist and the obtained scores had indicated that fMLF binding in FPR1 was more favorable than in FPR2 with scores −7.8 kcal mol^−1^ and −6.1 kcal mol^−1^, respectively. The binding of fMLF to FPR3, which shares 56% sequence identity with FPR1, is below detection limits [17]. The obtained homology model of FPR3 also exhibited many differences including residues in the binding site: (FPR1 to FPR3) F81R^2.60^, R84S^2.63^, K85V^2.64^, F102H^3.29^ and D284N^7.38^ (Figure 10B). The loss of fMLF binding can be attributed to the mutations K85V^2.64^ and R84S^2.63^ both of which had been shown to be important for binding. Moreover, F81R^2.60^ could also contribute to the lack of fMLF binding since hydrophobic properties were lost at the position where the F3 residue of the ligand is located. Furthermore, residues in the activation zone at positions 201 and 205, namely R205H^5.42^ and R201F^5.38^, were also found to have properties different from FPR1 indicating that the activation must be performed in another way than in the case of FPR1 and FPR2.

## Discussion

### The choice of CXCR4 as template structure

Our study is the first attempt, to our knowledge, to show changes in the molecular structure of FPR1 that occur upon agonist binding. The structure was constructed based on a novel template, the chemokine receptor CXCR4, belonging to the same γ branch of the phylogenetic tree of GPCRs as the formyl receptors. Molecular dynamics simulations were conducted including an all-atom model of the membrane. Because there are two structures of CXCR4 complexed with different antagonists we chose the one in which helices are not distorted by the presence of detergent used for crystallization. The structure with a small agonist (PDB id 3ODU) contains two detergent molecules between helices TM5 and TM6. They do not affect the binding of an antagonist IT1t to CXCR4 which occurs mainly to helices TM2, TM3 and TM7. In the case of FPR1 the experimental evidence is that TM5 participates extensively in the binding of agonists and antagonists. Therefore, we decided to use the structure of CXCR4 complexed with a cyclic peptide CVX15 which is also an antagonist of this receptor (PDB id 3OE0 [18]) in spite of its lower resolution 3.2 Å compared to 2.5 Å of the structure with IT1t.

### Comparison of structures based on rhodopsin and CXCR4 templates

**Figure 11:**
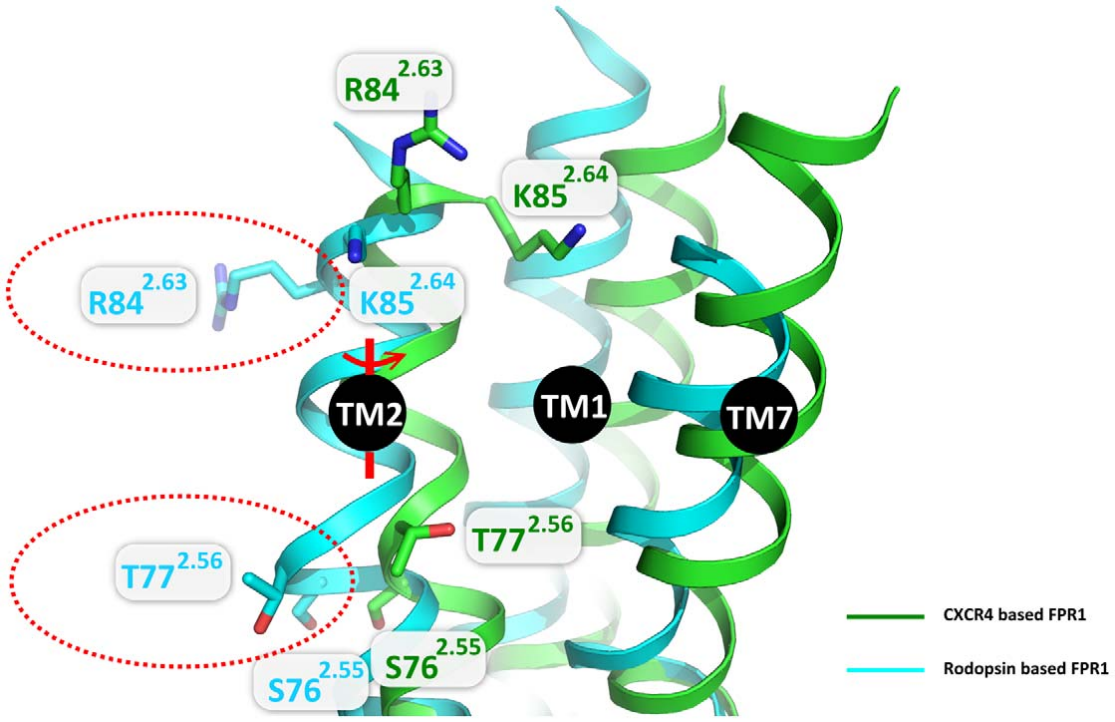
Comparison of FPR1 models constructed on different templates. A model based on rhodopsin is colored in cyan while that based on CXCR4 in green. Some residues in TM2 are shown in red dashed ellipses to exemplify differences between both models. A change of a template from rhodopsin to CXCR4 leads to the rotation about 100° of extracellular part of TM2 starting from S76^2.55^ and removal of a bulge at T77^2.56^.

Earlier modeling attempts of FPR1 [13]–[15] were all based on the rhodopsin template. There are several important differences between the rhodopsin and CXCR4 structures which can affect homology modeling and binding of ligands. First, the EC2 loop is outside the binding site of CXCR4 so there is much more space for binding of a ligand, and second, there is a bulge at the extracellular part of TM2 of rhodopsin (located at positions S76^2.55^ and T77^2.56^ of FPR1) which is not present in the CXCR4 structure. Using the CXCR4 template this part of TM2 is rotated about 100° compared to the rhodopsin template so that another part of TM2 is facing the binding site (Figure 11). In particular, residue R84^2.63^ which was predicted, based on the rhodopsin structure, to be outside the binding site can now interact with the ligand together with K85^2.64^. Interestingly, both these residues were predicted by Mills *et al.* [17] to strongly interact with ligands of FPR1. Additional confirmation of the obtained structure is the presence of a salt bridge between K85^2.64^ and D284^7.38^ which was proposed by Mills based on site-specific fluorescent photoaffinity labeling and mass spectrometry [17]. The mutual location of helices other than TM2 is also different in both templates so the binding site is dissimilar enough to prefer other ligand binding modes. The presence of the bulge in TM2 in rhodopsin could severely influence the structure and interactions in the binding site of homology models and it was one of major reasons for very poor docking results during modeling of complex of CXCR4 structure during GPCR Dock 2010 assessment [27].

According to the location of residues in contact with docked ligands, the binding pocket of FPR1 can be visually divided into two zones: the activation zone where the modified N-terminus of fMLF and tBocMLF is bound, and the binding zone where the ligand C-terminus is bound. The nearly symmetrical binding site of FPR1 enables reversed binding configurations of the agonist and antagonist and one cannot exclude that initially the ligands can bind in both ways with the N-terminus docked either to TM5 or to TM2. However, only one way can be appropriate for activation of the receptor. The receptor binding site is more spacious close to TM5 because this helix is located farther from the EC2 loop and this could be the reason for preferential docking of the tBoc moiety to this area as well as further loosening of the bonding between the C-terminus of the antagonist and TM2 during MD simulation. fMLF was stably bound to TM2 during the entire MD simulation. These findings are also supported by experimental data because fMLF shows higher binding affinity than tBocMLF in the case of native FPR1.

### Binding of tripeptide and tetrapeptide ligands

In the study of Movitz *et al.* [15] the authors identified a tetrapeptide of the ligand annexin A1, Gln^9^-Phe^12^ (Ac-QAWF), as the shortest sequence of annexin A1 which is still able to activate FPR1. They also modeled the structure of FPR1 based on the rhodopsin template and proposed a binding mode of this peptide so it was bound to both helices TM2 and TM5 and spanned across the entire binding site. Tripeptides such as fMLF are shorter than Ac-QAWF so the binding mode must be different. We found that the water molecules can bridge interactions between the N-terminus of the agonist and charged residues D106^3.33^, R201^5.38^ and R205^5.42^ and therefore they can participate in the activation process. Based on experimental results [28]–[31], four hydrophobic residues, namely F81^2.60^, V101^3.28^, F102^3.29^ and F291^7.43^, had been shown to be important for fMLF binding. Moreover, residues Y257^6.51^, K85^2.64^ and R84^2.63^ [17], [30] were also identified by mutagenesis to have a significant effect on FPR1 binding affinity, while D106^3.33^ [17], [31], R201^5.38^ and R205^5.42^ [30] were confirmed to be crucial for FPR1 activation. All these residues were found in close vicinity of the docked, optimized and simulated agonist fMLF.

During MD simulation the side chain of the M1 residue of the agonist went down toward the center of the receptor close to residues regarded to be crucial for activation of most of GPCRs including W254^6.48^ from the CWxP motif in helix TM6. This residue participates in the so called transmission switch, the action of which leads to rearrangements of residues in the central part of GPCRs and is a prerequisite for outward movement of the cytoplasmic part of helix TM6 (a recent review on the action of molecular switches in GPCRs can be found in [32]). Contrary to the agonist the side chain of residue M1 in tBocMLF was displaced toward the EC2 loop. There is a similarity of the location of the side chain of the first amino acid of fMLF and of the tetrapeptide Ac-QAWF. In both cases this side chain is located in close vicinity of W254^6.48^. The tetrapeptide was manually docked in Movitz *et al.* work [15] to preserve interactions with residues in TM3 and TM5 known to participate in activation. In our simulations a hydrogen bond between a formyl group and S287^7.39^ was created during all MD simulation of FPR1 with agonist. Such binding could also contribute to a small movement of helices TM3 and TM7 (Figure 8A) and facilitated changing of a rotamer switch of Y301^7.53^.

### Role of water molecules in ligand binding

Water molecules were found to be important also in a recent paper of Vanni *et. al.* [33] in 800 ns MD simulation of β_2_-adrenergic receptor. They bridged interactions between agonists and serine residues located in TM5 while the ligands were closely bound to D113^3.32^ in TM3 with their protonated amine group. Displacement of these water molecules may be a step towards the activation of the receptor because it was found that the binding site of β_2_-AR is shrinking during activation [34]. Two water molecules were also found to bridge the interaction between phenolic hydroxyl groups of antagonists and the side chain of H(6.52) in three crystal structures of opioid receptors μOR, δOR and κOR. Identical arrangements of these water molecules in three different receptors suggest that their presence is crucial to stabilize the antagonist and possibly they participate in receptor activation when an agonist is bound. In our earlier papers on activation of opioid receptors [35]–[37] we postulated, based on MD simulations, that antagonists can bind to residues in TM3, namely D(3.32) and Y(3.33), but agonists can swap from Y(3.33) to H(6.52) in helix TM6 and such change of location is probably one of the first activation steps. Since no structures of opioid receptors with agonists are available, this hypothesis still needs to be validated. Possibly, during activation these water molecules are displaced and the agonist can bind directly to H(6.52). This can shrink the binding site and facilitate rearrangement of residues of the central part of the receptor which constitutes a part of the transmission switch. This switch was previously called the rotamer toggle switch and was linked only to residue W(6.48), however, the suggested action of this switch was not confirmed by later crystal structures of GPCRs with agonists.

**Figure 12:**
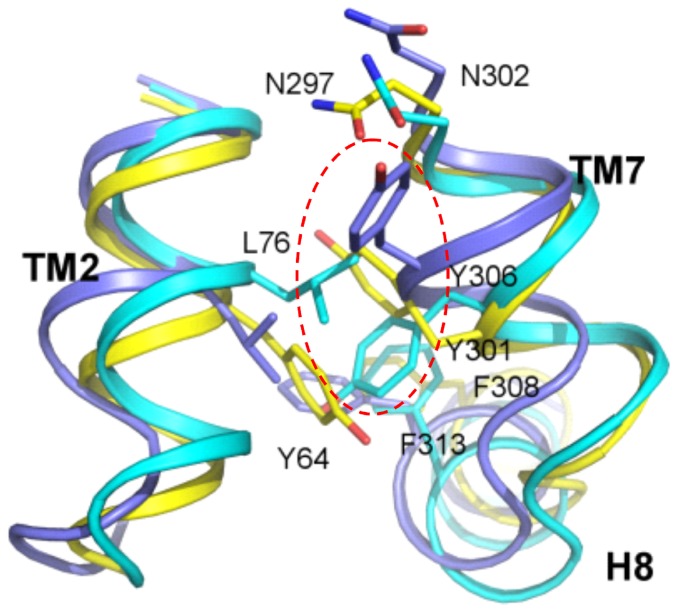
A comparison of rotamers of residue Y7.53 from NPxxY motif in different receptor structures. FPR1 with agonist bound in yellow (Y301), inactive rhodopsin (PDB id 1GZM) in light blue (Y306), activated rhodopsin (PDB id 2X72) is dark blue (Y306). Dashed red line encircles the three abovementioned tyrosines.

In a recent structure of the muscarinic receptor M2 [38] there is an aqueous channel extending from the extracellular surface into the transmembrane core with well-ordered water molecules. This channel is interrupted by a layer of hydrophobic residues located in helices TM2, TM3 and TM6 close to residue Y(7.53) in the NPxxY motif. Although the Tyr toggle switch is in an active state (i.e. the side chain of Y(7.53) is directed toward the receptor center contrary to the rhodopsin structure in which it is directed toward the cytoplasmic helix H8) [32] there is no hydrogen bond network linking Y(7.53) with N(7.49). Possibly, after the action of the transmission switch in the muscarinic M2 receptor the channel will be rearranged and an extended hydrogen bond network will connect both sides of the receptor to enable final stages of receptor activation. Such an extended network of hydrogen bonds involving water molecules crossing the hydrophobic barrier was found recently in the structure of constitutively active rhodopsin [39]. In the model of FPR1 we also found an extended network of hydrogen bonds (Figure 9). Such network was broken at residue Y301^7.53^ since it created a π-π stacking interaction with Y64^2.43^. What is interesting, after the Tyr switching the interaction of Y301^7.53^ with Y64^2.43^ is still maintained while there is a space in the receptor center for water molecules coming towards the receptor center in larger amounts (Figure 8C). We also compared rotamers of the residue Y7.53 in different GPCRs and found that there were only two positions of this switch - hence it was called toggle. One position was found in fully inactive rhodopsin (eg. PDB id 1GZM, it is additionally bound to F313 in H8) and the second in activated rhodopsin (PDB id 2X72) and also in the crystal structures of other GPCRs even with antagonists and inverse agonists bound. Here, we present the third possibility for the rotamer of Y7.53 (Figure 12). Such position of the Y7.53 residue may be too unstable to be found in the crystal structure but is taken by the receptor temporarily to introduce water to the receptor center. It is also possible that this position is specific to FPRs.

To resolve unanswered questions of activation details and ligand docking as well as ligand selectivity the MD simulations in a microsecond time scale have to be conducted, preferably based on the solved crystal structures of FPRs. Knowledge of these structures and the activation processes initiated by binding of the diverse ligands will lead to better understanding of mechanisms of action of these highly elusive receptors and also to a design of safer and more efficient drugs.

## Methods

### Homology modeling and refinement of FPR1

The homology models of FPR1 were obtained by Modeller 9v8 [40] using the crystal structure of chemokine receptor type 4 (CXCR4, PDB id 3OE0) [18] which shares the highest homology (31.0% identity, 53.8% similarity) with FPR1 according to Discovery Studio Visualizer [41]. Since the region corresponding to helix H8 at cytoplasmic side of CXCR4 is unfolded in the crystal, the crystal structure of human β_2_-adrenergic receptor [42] (PDB id 2RH1) was used as the second template for the H8 regions of FPR1. The sequence alignments (Figure S5 in File S1) were performed automatically in MUSCLE [43] and adjusted manually in Discovery Studio Visualizer [41] for proper aligning of conserved motifs and disulfide bridge. The 1500 models of initial FPR1 receptor were generated in Modeller with fully annealed protocol, and the optimal model was chosen according to DOPE (Discrete Optimized Protein Energy) score [44]. Low homology regions of loops between transmembrane helices were constructed with loop refinement protocol in Modeller and the lowest DOPE score model from 1000 generated models was selected for further study. To obtain the proper orientation of the receptor in the membrane the refined model of FPR1 was aligned with CXCR4 crystal structure (PDB id 3OE0) taken from OPM (Orientations of Proteins in Membranes) database [45]. The hydrogen atoms were added to the FPR1 structure according to the physiology pH environment. To remove unfavorable steric contacts and to release strain among amino acid residues the model was submitted to Prime (Schrödinger 2011 suite) [46] for backbone-constrained truncated-Newton minimization refinement, using the OPLS_2005 force field [47] and implicit membrane model.

### Receptor model equilibration in explicit membrane

Using the builder tool for Desmond [48] in Maestro 9.2 program [49] the FPR1 model was embedded into pre-equilibrated POPE (1-palmitoyl-2-oleoyl-*sn*-glycero-3-phosphoethanolamine) lipid bilayer solvated with water and NaCl to make the system neutral and set ionic strength 0.15 M. The total number of atoms was approximately 54,000 including 28 Na^+^ and 40 Cl^−^ ions, about 10,000 water molecules, and 161 POPE molecules. The periodic box dimensions were about 6.8 nm×7.2 nm×9.4 nm. Equilibration of the system was performed at constant pressure and temperature (NPT ensemble; 310 K, 1 bar) and Berendsen coupling [50] scheme with one temperature group. All bond lengths to hydrogen atoms were constrained using M-SHAKE [51]. Van der Waals and short-range electrostatic interactions were cut off at 1.0 nm. Long-range electrostatic interactions were computed by the particle mesh Ewald (PME) summation scheme [52]. A RESPA (time-reversible reference system propagator algorithm) integrator [53] was used with a time step of 1.6 fs. Long-range electrostatic interactions were computed every 4.8 fs. Harmonic positional restraints on the protein were tapered off linearly from 10 to 1 kcal mol^−1^ Å^−2^ over 16 ns.

### Ligand preparation and docking

Both ligands fMLF and tBoc-MLF were built in Maestro program. Ligand preparation utility was used to optimize the geometry of initial structures. Systematic conformational search was performed in MacroModel [54] and the top five conformers with the lowest potential energy were kept for docking. The docking procedure was performed using Glide [55], [56] (Schrödinger 2011 suite). Ligand molecules were initially placed in the binding pocket with a random pose. Cubic boxes centered on the ligand mass center with a radius 1.5 nm for both fMLF and tBocMLF defined the docking binding regions. Flexible ligand docking was executed in all cases. Twenty poses per ligand out of 2000 were included in post-docking energy minimization. Top three scored poses were similar to each other, thus only one the best scored pose per each ligand was chosen as the initial structure for MD simulations.

### Molecular Dynamics

To obtain the non-standard residues (-CHO and tBoc-) the force field parameters for MD simulation, the partial atomic charges for the ligands were obtained in GAUSSIAN 09 program [57] via obtained Hartree-Fock 6–31G* electrostatic potential (ESP) and then using the fitting procedure performed by the R.E.D. tool [58]. The membranous system was built and equilibrated as mentioned above. Nine 100 ns MD simulations with 1.0 kcal mol^−1^ Å^−2^ harmonic restraints on backbone of TM regions were conducted employing CHARMM36 full-atom force field [59]. Three simulations for Apo-FPR1 as well as three simulations per each of its complexes with agonist fMLF and antagonist tBocMLF. Using harmonic restraints restricts sampling to the neighborhood of the initial model and prevents deterioration of the homology model which is a result of insufficient accuracy of current force fields [60]. Data analysis was done using Desmond utilities and the molecular figures were made in VMD [61] and Pymol [62].

## Supporting Information

File S1 **Contains Figures S1, S2, S,3 S4, S5.**
(PDF)

Movie S1 **A bridging of a hydrogen bond between W254^6.48^ and N108^3.35^ by water molecule in agonist fMLF-FPR1 complex.** During MD simulations a water molecule which was initially located between R205^5.42^ and the formyl group of agonist fMLF diffuses down to the receptor center and bridges an interaction between W254^6.48^ and N108^3.35^.
(WMV)
