# Supplementary material for: The Role of Water in Activation Mechanism of Human N-Formyl Peptide Receptor 1 (FPR1) Based on Molecular Dynamics Simulations
Source: PLoS One. 2012 Nov 26;7(11):e47114. doi: 10.1371/journal.pone.0047114 (PMC3506623; doi:10.1371/journal.pone.0047114)
Supplement: File S1 — Contains Figures S1, S2, S,3 S4, S5. (PDF) [file pone.0047114.s001.pdf]

The role of water in activation mechanism of human N-formyl Peptide Receptor 1 (FPR1) based on molecular dynamics simulations

Shuguang Yuan[1,2], Umesh Ghoshdastider[1], Bartosz Trzaskowski[3], Dorota Latek[1], Aleksander Debinski[3], Wojciech Pulawski[3], Rongliang Wu[1], Volker Gerke[4], Slawomir Filipek[3]*

Supplementary material

Figure S1. The root mean squares deviation (RMSD) plots for FPR1 backbone in exemplary 100 ns MD simulations for each analyzed system. The colors are the following: FPR1 Apo in black, fMLF/FPR1 in green, and tBocMLF/FPR1 in blue.

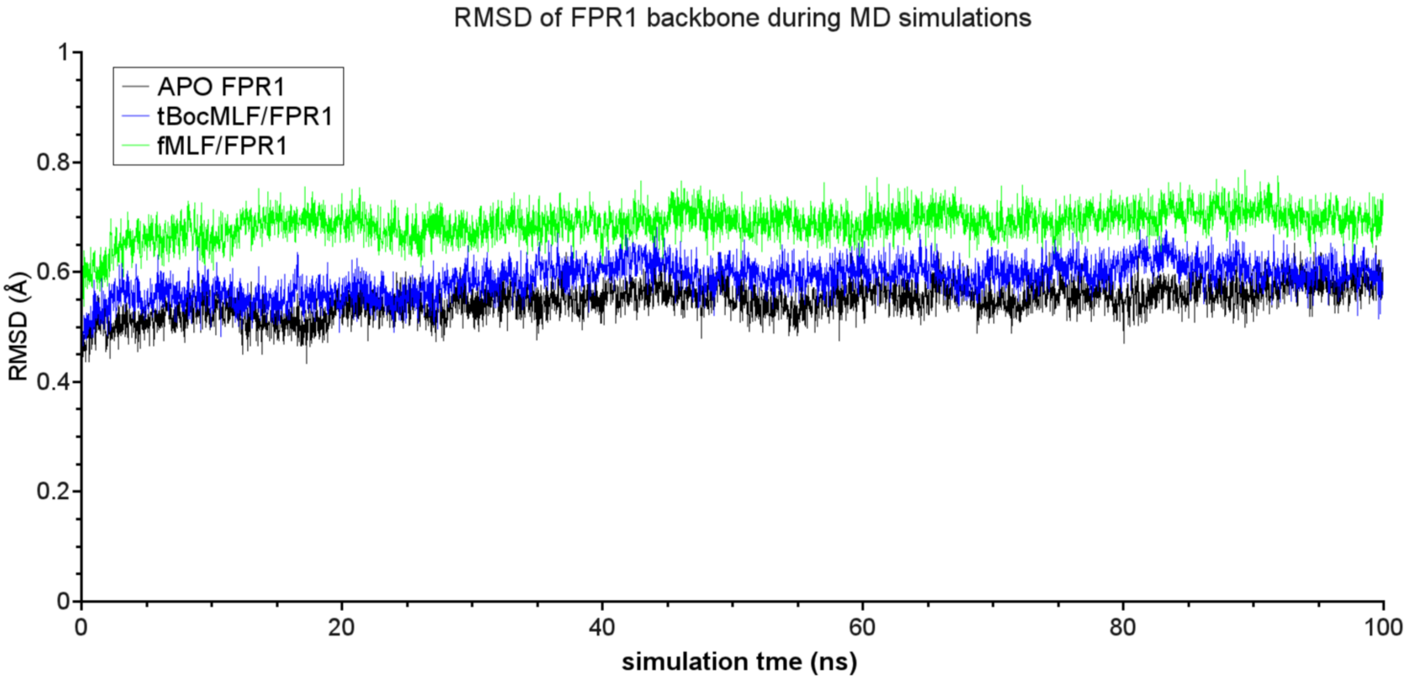

Figure S2. A distance between D106$^{3.33}$ to arginine residues in TM5. (**A**) to R201$^{5.38}$ (**B**) to R205$^{5.42}$**.** Top panels - Apo FPR1; middle panels - a complex with antagonist tBocMLF; bottom panels - a complex with agonist fMLF. The distances were measured between CG atom of D106$^{3.33}$ and CZ atom of arginine residues.

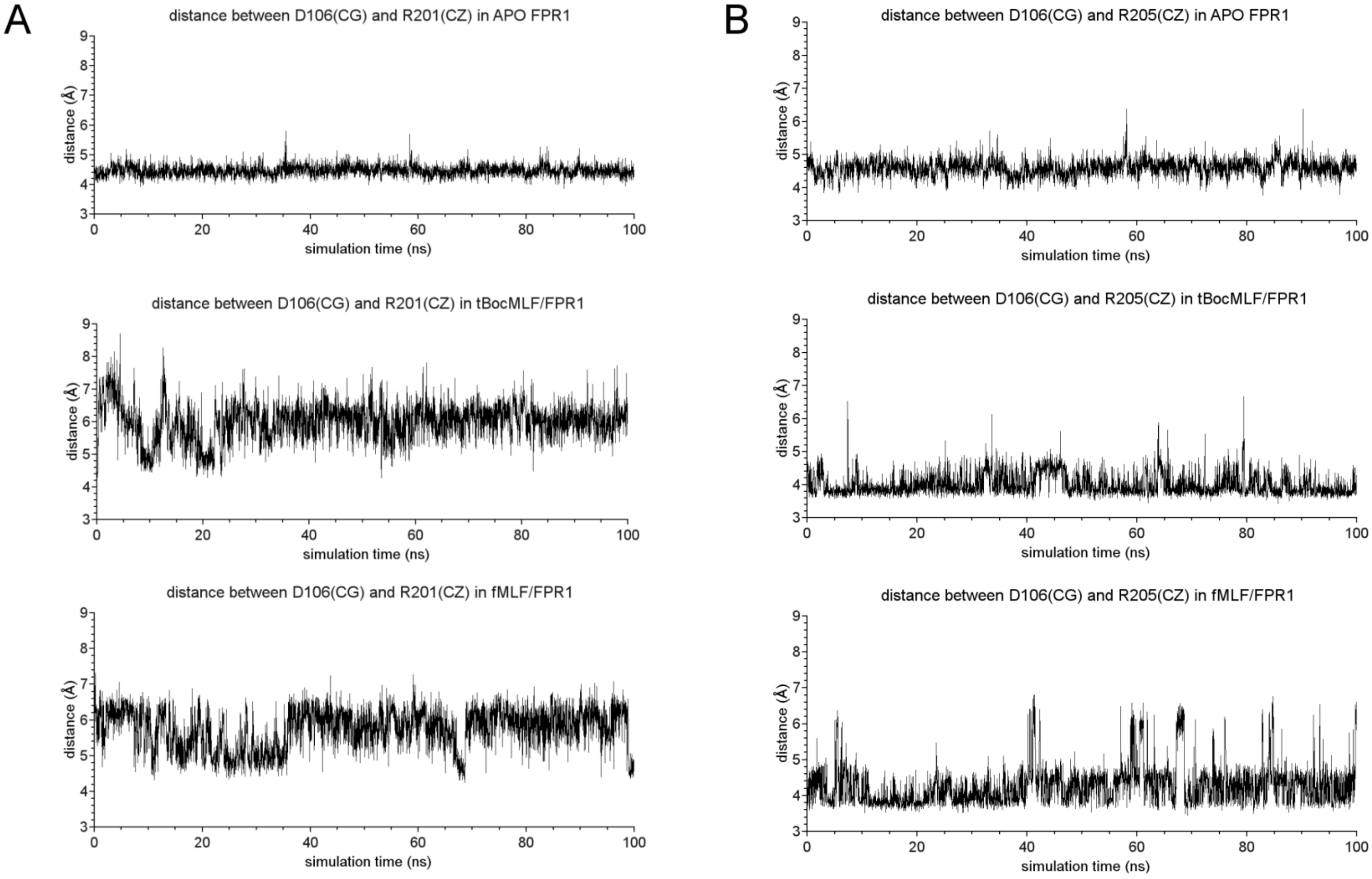

Figure S3. The effect of bridging water molecules in agonist-receptor complex. (**A**) Bridging a hydrogen bond between W254$^{6.48}$ and N108$^{3.35}$; (**B**) and between N297$^{7.49}$ and Y301$^{7.53}$. For comparison the analogous distances in exemplary simulations of Apo FPR1 (first panels) and in the complex with antagonist tBocMLF (second panels) are shown where the hydrogen bonds (A) and (B) are stable during the whole simulation. In the complex with fMLF (three bottom panels) the hydrogen bond was bridged temporarily in case (A) but stable in case (B).

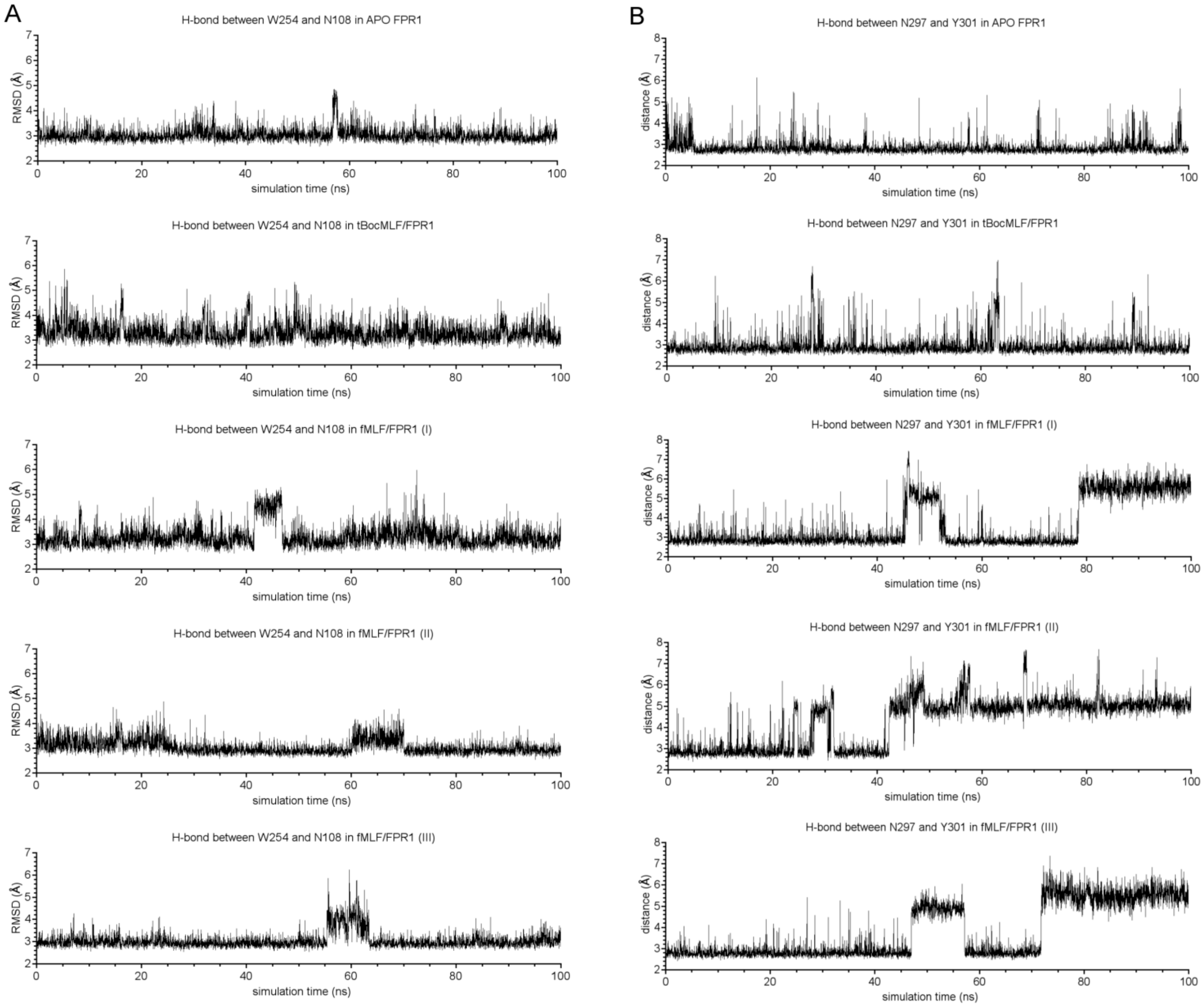

Figure S4. Forming of hydrogen bond between formyl group of fMLF and $S287^{7.39}$. This bond was formed in each of three 100 ns simulations of FPR1 with agonist.

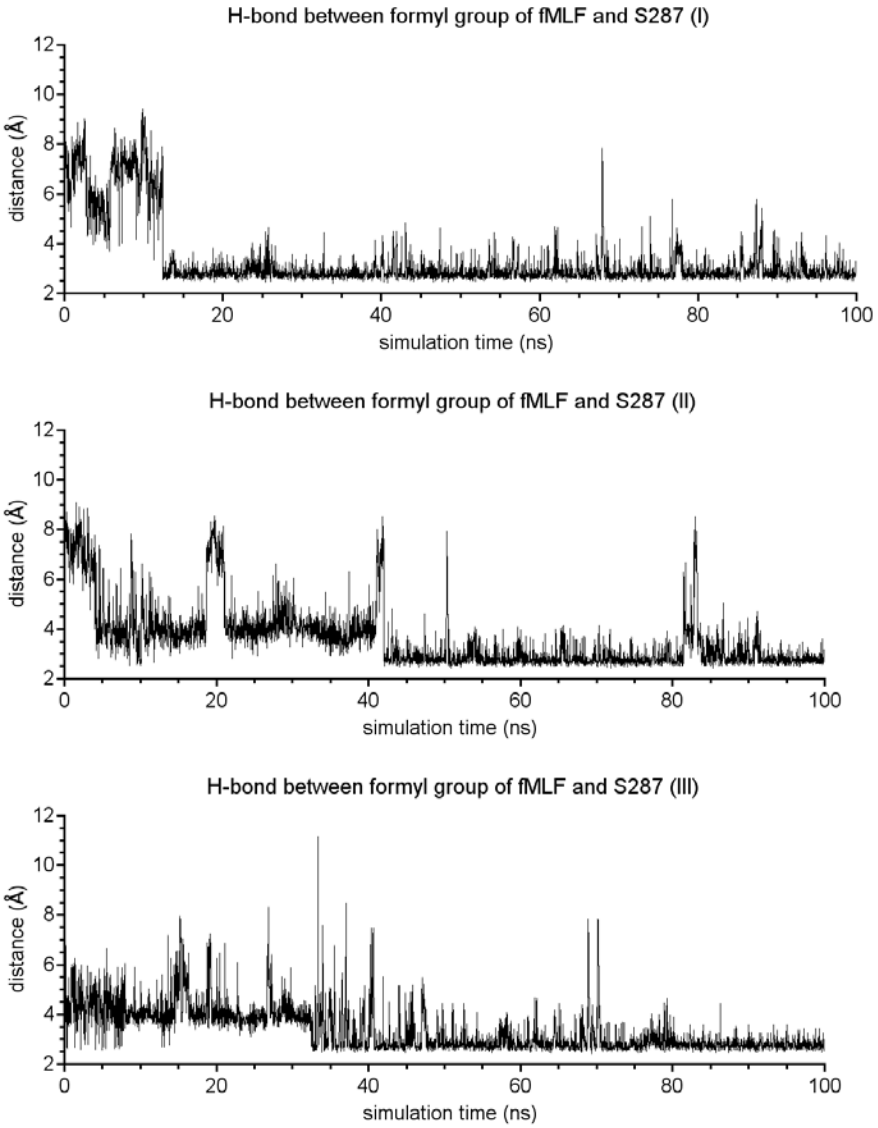

Figure S5. Sequence alignment for FPR1 homology modeling. The seven TM regions were built based on crystal structure of CXCR4 (PDB id 3OE0) and the helix H8 was generated based on β_2-adrenergic receptor (PDB id 2RH1). Identical and similar residues in alignment are marked with blue color, darker and lighter, respectively. The most conserved residues in each helix (x.50) as well as the conserved motifs are encircled with green ellipses.

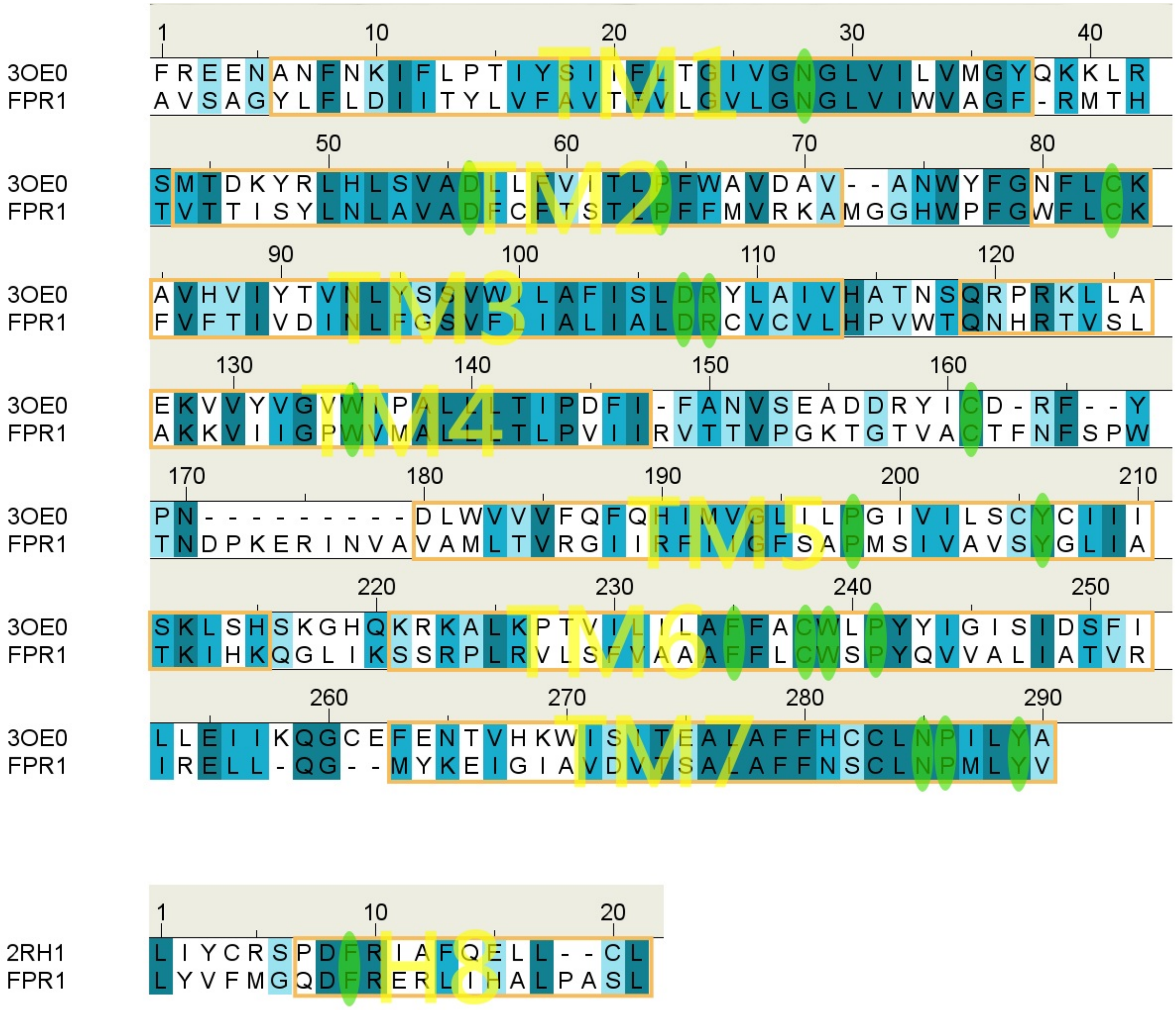

Movie M1. A bridging of a hydrogen bond between W254$^{6.48}$ and N108$^{3.35}$ by water molecule in agonist fMLF-FPR1 complex. During MD simulations a water molecule which was initially located between R205$^{5.42}$ and the formyl group of agonist fMLF diffuses down to the receptor center and bridges an interaction between W254$^{6.48}$ and N108$^{3.35}$.
